# Modeling of Bead-on-Plate Laser Beam Melting Using Innovative Laser with a Single-Mode Core Surrounded by a Multimode Ring

**DOI:** 10.3390/ma19071423

**Published:** 2026-04-02

**Authors:** Marcin Kubiak, Zbigniew Saternus, Tomasz Domański, Michał Urbańczyk, Krzysztof Talaśka, Dominik Wilczyński, Dominik Wojtkowiak

**Affiliations:** 1 Faculty of Mechanical Engineering, Czestochowa University of Technology, 42-200 Częstochowa, Poland; zbigniew.saternus@pcz.pl (Z.S.); tomasz.domanski@pcz.pl (T.D.); 2 Łukasiewicz—Upper Silesian Institute of Technology, 44-100 Gliwice, Poland; michal.urbanczyk@git.lukasiewicz.gov.pl; 3 Faculty of Mechanical Engineering, Poznan University of Technology, 61-138 Poznań, Poland; krzysztof.talaska@put.poznan.pl (K.T.); dominik.wilczynski@put.poznan.pl (D.W.); dominik.wojtkowiak@put.poznan.pl (D.W.)

**Keywords:** SM–MMR systems, core beam, ring beam, single-mode laser, multimode laser, thermal phenomena

## Abstract

Laser beams are widely used in heat treatment and welding processes. Due to limitations of a single beam, hybrid solutions with dual beams have been developed. One of the newest approaches uses a single-mode laser as the core of the heat source combined with a surrounding multimode ring beam. The aim of this work is to develop a mathematical and numerical model of the power density distribution for such a combined laser source. The power distribution is described using a cylindrical-power-involution model. The model is applied to simulations of transient thermal phenomena during bead-on-plate laser melting of 4 mm thick S355 steel plates. The computational domain represents surface melting without a joint gap, and heat transfer occurs by conduction into a single plate. The predicted fusion zone and heat-affected zone are compared with macroscopic cross-sections of experimental bead-on-plate tracks. Good agreement confirms the suitability of the proposed dual-beam model for bead-on-plate laser processing of structural steel.

## 1. Introduction

Laser beam processing has evolved substantially since the introduction of early solid-state lasers such as lamp-pumped Nd:YAG systems, which provided limited beam quality and modest penetration capabilities [1]. Subsequent generations of diode-pumped Nd:YAG lasers and disk lasers improved efficiency and beam stability, yet still delivered predominantly Gaussian beam profiles that restricted process flexibility [2,3,4]. With the advent of high-power fiber lasers, industrial laser processing benefited from excellent beam quality, high brightness, and the ability to produce deep keyhole penetration [5,6]. However, the sharply peaked intensity of single-mode fiber lasers introduced challenges including keyhole oscillations, porosity formation, and narrow molten pools sensitive to joint gap variations.

To counter these limitations, various beam-shaping strategies have been developed. Dual-beam systems—using either two single-mode lasers arranged in tandem or a combination of high-quality and defocused beams—demonstrated improved stability and reduced porosity [7,8]. Hybrid laser–arc welding [9,10] provided additional tolerance to joint fit-up but introduced significant process complexity. Another path has been the use of multimode or ring-shaped beams, which distribute heat over a broader area, thereby stabilizing the keyhole and diminishing the extreme thermal gradients typical for single-mode beams [11,12,13].

An important milestone in the evolution of laser processing was the widespread industrial adoption of diode-pumped solid-state fiber lasers (DPSSLs). The transition from lamp pumping to semiconductor diode pumping dramatically increased electrical-to-optical efficiency, reduced thermal loading of the gain medium, and enabled stable, high-brightness operation. The fiber geometry provided excellent heat dissipation and allowed the generation of nearly diffraction-limited single-mode beams at multi-kilowatt power levels. At the same time, advances in cladding-pumped architectures enabled the production of high-power multimode fibers capable of delivering large mode areas suitable for broader, more uniform beam profiles. This combination of diode-pumped gain efficiency and flexible fiber design laid the technological foundation for modern welding systems in which both single-mode core beams and multimode ring beams can be generated, combined, and shaped with high precision [14,15,16]. Consequently, diode-pumped solid-state fiber technology represents the essential stepping stone that made possible today’s integrated single-mode/multimode coaxial irradiation strategies.

In recent years, a new generation of optically integrated heat sources has emerged: single-mode core/multimode ring (SM–MMR) systems [12,13,17]. These sources combine a central single-mode beam responsible for penetration with a surrounding multimode ring that supplies distributed energy, effectively stabilizing the molten pool. Unlike earlier dual-beam or hybrid configurations, SM–MMR systems provide a coaxial, monolithic beam profile with tunable energy sharing between the core and ring. The result is enhanced process robustness, improved weld surface quality, and broader processing windows for materials susceptible to porosity or instability. Literature reports increasingly point to significant benefits in keyhole stability, melt pool shape control, and suppression of spatter, making SM–MMR irradiation one of the most promising directions for advanced laser processing. Recent studies demonstrate that such coaxial dual-profile beams enable finer adjustment of weld geometry by controlling the relative power fraction between the core and the ring, the ring diameter, and the beam divergence. Compared to conventional solid-state lasers with Gaussian profiles, these systems offer a more distributed heat input that can be tailored to suppress keyhole collapse, ensure uniform melting, and enhance the stability of deep-penetration welding. As a result, the single-mode core/multimode ring architecture is emerging as a promising direction for next-generation laser processing applications [17,18,19].

SM–MMR beam shaping is particularly attractive for welding critical structural components used in civil engineering, such as thick-walled steel plates, bridge elements, stiffeners, and high-strength structural steels. These applications require high process robustness, controlled penetration, and reduced susceptibility to porosity and lack of fusion, especially under industrial fit-up tolerances [20,21]. The distributed heat input provided by SM–MMR beams enables wider molten pools and moderated thermal gradients, which can reduce residual stresses and improve weld quality in structural steels commonly used in construction, including S355 and higher-strength grades [21,22]. Compared with conventional single-mode Gaussian beams, coaxial core–ring irradiation offers improved tolerance to surface condition variations, joint misalignment, and thickness transitions frequently encountered in large-scale civil engineering structures [21,22,23].

Numerical simulation of laser melting processes relies heavily on accurate mathematical representations of thermal phenomena [12,24,25,26,27,28]. The complexity of weld pool formation—spanning conduction, convection, phase transitions, and fluid flow—requires simplified yet physically meaningful descriptions of the heat source [12,25,26,27,28,29,30,31,32,33,34]. The literature features several canonical models, like Gaussian heat source models [29,30], originating from Rosenthal’s analytical solutions, that are widely used for representing highly focused laser beams. Single-mode fiber lasers with near-diffraction-limited beam quality are approximated well by Gaussian or multi-Gaussian distributions. These models capture the steep intensity gradients typical of deep-penetration keyhole welding. Top-hat and cylindrical heat sources [32,33] are often applied to multimode or defocused beams, where the intensity is more uniformly distributed. Variants using polynomial or power-involution functions allow smooth transitions between flat-top and Gaussian-like profiles, offering flexibility for modeling ring-shaped beams or broadened irradiance distributions. Goldak’s double-ellipsoid model [34], originally developed for arc melting, remains one of the most influential volumetric heat source representations in computational welding mechanics. Its ability to represent asymmetric heat deposition in front of and behind the heat source makes it suitable for hybrid processes and for approximating the combined action of a laser and arc, or multiple laser components. Combined and composite heat-source models, including superpositions of Gaussian beams, Gaussian–top-hat combinations, or Gaussian cores surrounded by lower-intensity envelopes, have been increasingly applied to simulate dual-beam and ring-beam laser welding [12,29,30,32]. In particular, analytical multi-component models enable representation of single-mode cores embedded within multimode rings, making them well suited for SM–MMR process simulation.

Across the literature, the choice of heat source model depends crucially on the beam profile, penetration mechanism, and the degree of computational complexity acceptable for the simulation [31,32,33,34]. Accurate modeling of SM–MMR systems therefore requires formulations capable of representing both the concentrated core energy and the distributed ring envelope [13].

Although the advantages of single-mode core/multimode ring material melting have been demonstrated experimentally, a consistent mathematical and numerical framework for representing such heat sources is still lacking. Existing analytical models are typically tailored either to highly concentrated beams or to broad multimode distributions [12], but few studies systematically integrate both within a unified representation.

From the modeling perspective, most existing numerical approaches rely on Gaussian, multi-Gaussian, or Goldak-type volumetric sources, which do not explicitly represent the coaxial redistribution of power characteristic of SM–MMR beams [24,25,26,27,28,29,30]. Consequently, there is a need for analytical formulations capable of simultaneously representing the highly concentrated penetration-driving core and the stabilizing multimode ring. The model proposed in this work addresses this gap by introducing a composite cylindrical-power-involution formulation tailored to SM–MMR heat sources, enabling improved prediction of fusion zone geometry and heat-affected zone dimensions for structural steel processing.

The purpose of this work is therefore to develop and evaluate mathematical models of the composite power density distribution characteristic of SM–MMR heat sources. Cylindrical-power-involution distribution [33] is employed to construct flexible representations of the combined core and ring components. These heat source formulations are implemented in numerical simulations of bead-on-plate laser melting of S355 steel plates, enabling detailed prediction of temperature fields, fusion zone geometry, and heat-affected zone extent.

The numerical results are compared with macroscopic cross-sections of experimentally produced bead-on-plate tracks to assess the fidelity of the core and the ring heat-source models and to determine their applicability to the prediction of thermal phenomena in SM–MMR laser processing. The findings contribute to a deeper understanding of the thermal behavior governing advanced dual-beam laser melting and provide modeling tools useful for process optimization and industrial implementation.

## 2. Experimental Research

**Figure 1:**
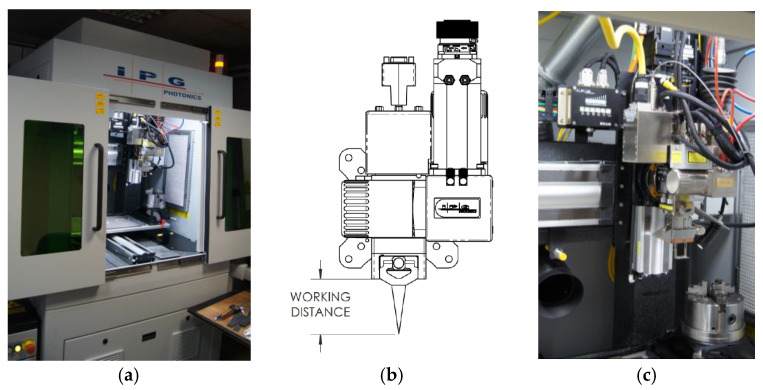
Solid-state fiber laser pumped with semiconductor laser diodes IPG YLS-2000/4000-SM-AMB (Marlborough, MA, USA): (**a**) working area, (**b**) schema of the welding head, (**c**) welding head in the working area.

The research is conducted at Łukasiewicz Upper Silesian Institute of Technology using an IPG MultiAxis laser welding station (Marlborough, MA, USA) equipped with a solid-state fiber laser pumped by semiconductor laser diodes (Figure 1). The active medium is an ytterbium-doped fiber (YLS). This is a single-mode (SM) laser with two adjustable beam modes (AMB).

**Figure 2:**
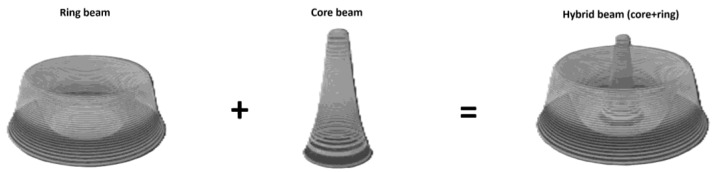
Schematic diagram of the multi-beam power distribution in the IPG YLS-2000/4000-SM-AMB laser source.

Bead-on-plate laser melting is carried out on steel sheets made of S355J2 steel with a thickness of 4 mm. The first, central beam (core beam) in the YLS-2000/4000-SM-AMB laser source (IPG Photonics Corporation, Marlborough, MA, USA) has a single-mode distribution and a maximum power of 2 kW, while the outer beam (ring beam) has a multimode distribution and a maximum power of 4 kW (Figure 2). The central and outer beams can operate simultaneously in the same location and at the same time, or they can operate independently of each other. The power density changes for both beams can be adjusted smoothly and individually for each laser beam. The YLS–2000/4000–SM-AMB laser source (IPG Photonics Corporation, Marlborough, MA, USA) can operate in both continuous and pulsed modes, with a wavelength of 1070 nm.

In order to determine the correct technological parameters of the process, a number of tests are performed for laser heating with a single core beam, a single ring beam and a combined power of the core and ring beams for various heat source travel speeds and different heat source powers. The central beam (core beam) had a diameter of 30 µm, while the outer beam (ring beam) had a diameter of 200 µm. The beams were focused on the surface of the welded sheets (z = 0).

**Figure 3:**
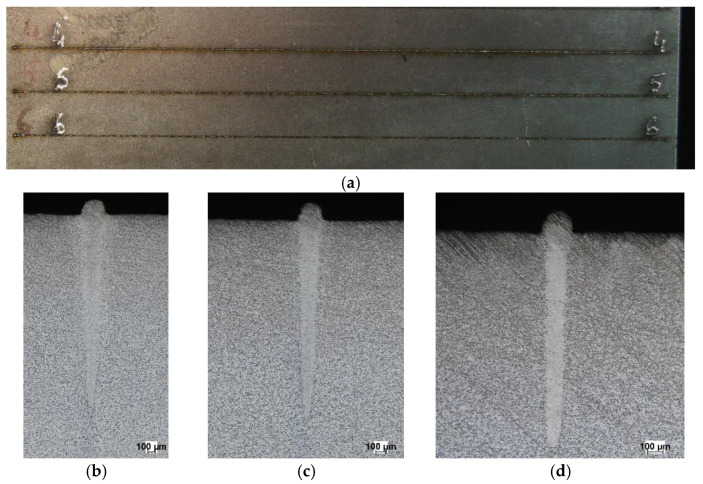
Macroscopic pictures of melted plates: (**a**) seam at the top surface and cross-section of the melted zone; (**b**) test no. 4, (**c**) test no. 5, (**d**) test no. 6.

**Figure 4:**
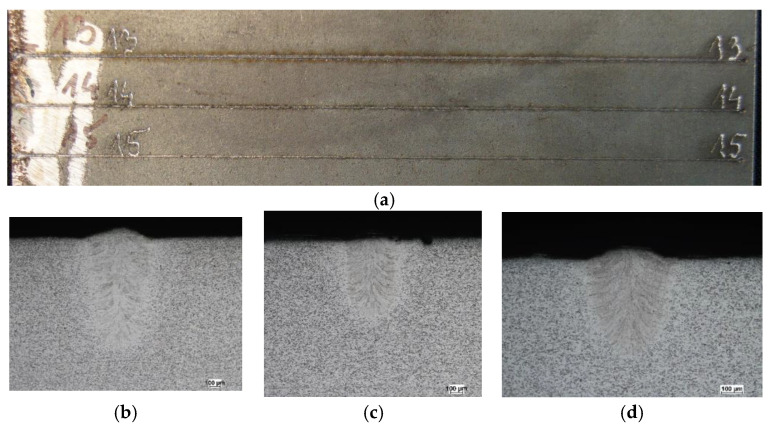
Macroscopic pictures of melted plates: (**a**) seam at the top surface and cross-section of the melted zone; (**b**) test no. 13, (**c**) test no. 14, (**d**) test no. 15.

**Figure 5:**
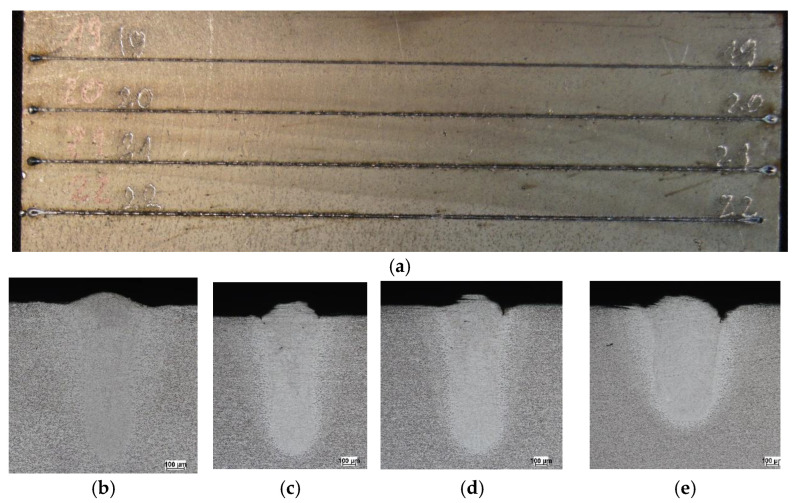
Macroscopic pictures of melted plates: (**a**) seam at the top surface and cross-section of the melted zone; (**b**) test no. 19, (**c**) test no. 20, (**d**) test no. 21, (**e**) test no. 22.

Table 1 presents selected technological parameters that correspond to Figure 3, Figure 4 and Figure 5, which show the melting seam on the top surface and in the cross-section of a plate melted with a core source (Figure 3), a ring source (Figure 4) and a hybrid core + ring source (Figure 5), respectively.

In core-beam melting (Figure 3), a very narrow fusion zone and heat-affected zone can be observed, with relatively deep penetration into the material. In the case of ring-beam melting (Figure 4), a wide fusion zone is obtained with low material penetration. Combining two sources cooperating in the weld pool (Figure 5) allowed for a wider fusion zone while maintaining material penetration at the same level as the core beam. It should be noted that this result was achieved at an increased heat source travel speed.

## 3. Modeling of the Heat Source

During laser beam processing, the penetration depth of the radiation in metallic materials is extremely small, typically on the order of 10^−4^–10^−5^ cm. As the surface rapidly absorbs the incident energy, the material enters a vaporization regime, creating a narrow cavity filled with ionized metal vapor—commonly referred to as a keyhole [24,28]. Within this cavity, the laser energy couples efficiently with the plasma and is subsequently conducted to the surrounding metal, generating and sustaining the molten pool. This melting phenomenon is generally described using a volumetric representation of the heat source. In such models, the laser power distribution is frequently approximated by a radial Gaussian profile, while the keyhole itself is idealized as a cylindrical or tapered (truncated-cone) volume.

### 3.1. Analytical Model of Single-Mode Core Heat Source

A universal cylindrical-involution-normal (CIN) model is adopted in this paper, according to the following formula [33]:

(1) $$Q(r,z)=\frac{kK_{z}Q_{L}}{\pi \left(1-e^{\left(K_{z}s\right)}\right)}e^{-\left(kr^{2}+K_{z}z\right)}\left(1-u\left(z-s\right)\right)$$

where *Q* is the laser beam power, *η_L_* is laser efficiency, *r*_0_ is a beam radius, $r=\sqrt{x^{2}+y^{2}}$ is a current radius, $K_{z}=3/s$ is a heat source power exponent, $k=3/r_{0}^{2}$ is a beam focus coefficient, *s* is the heat source beam penetration depth, and u(z−s) is a Heaviside function.

**Figure 6:**
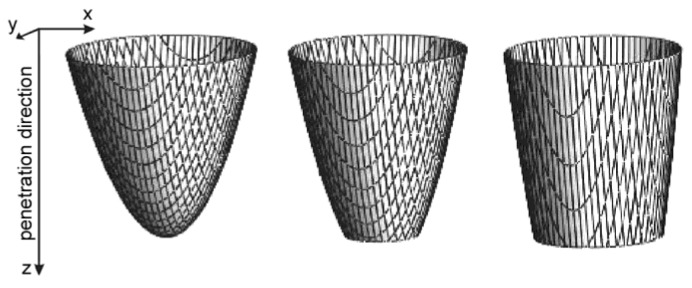
CIN heat source volume shape.

**Figure 7:**
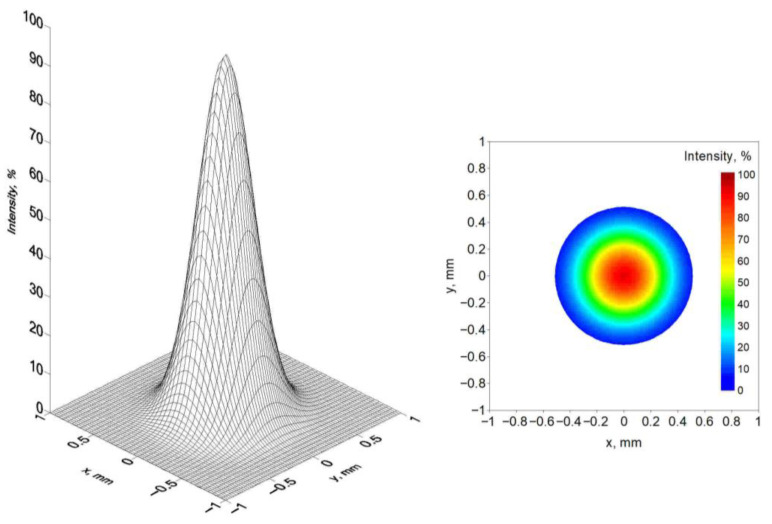
Exemplary percentage CIN heat source intensity distribution.

This heat source model, by changing *s*, *k* and *K_z_* parameters, allows for modeling of a variety of concentrated heat source shapes with exponential decreases in heat source energy with material penetration depth. Depending on the *s* factor, the “keyhole” can be considered as a paraboloid when workpiece thickness is greater than or equal to *s* or a truncated paraboloid when *s* is greater than the thickness (Figure 6). Exemplary laser beam heat source distribution at the top surface of the workpiece is presented in Figure 7.

### 3.2. Analytical Model of Multimode Ring Heat Source

**Figure 8:**
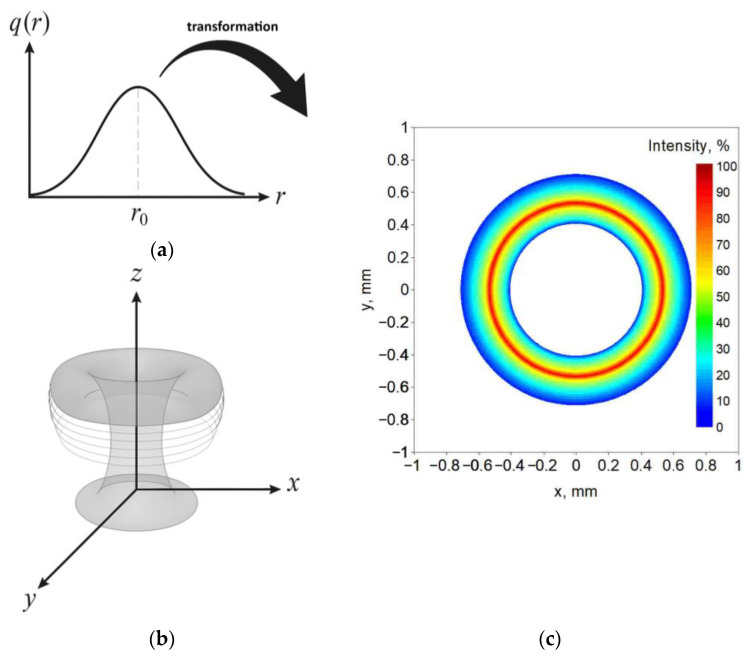
Construction of the ring-shaped heat source by rotation of a radial Gaussian (CIN) profile around the center of the laser spot: (**a**) one-dimensional CIN distribution *q*(*r*) along the radius, (**b**) three-dimensional power density field obtained by revolving *q*(*r*) about the z-axis, (**c**) power intensity distribution of a ring heat source.

The analytical model of the ring is based on the model described by Equation (1). The intensity distribution is based on the rotation of the CIN model around the center of the heat source spot (Figure 8).

**Figure 9:**
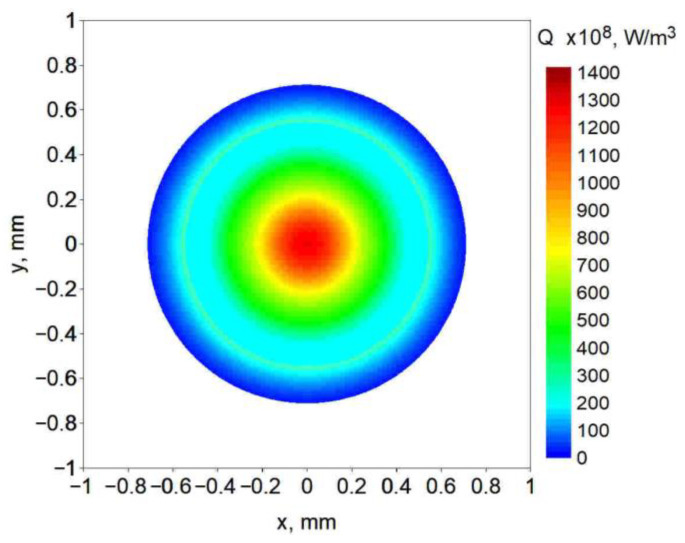
Volumetric power of laser beam heat source composed of single-mode core and multimode ring.

The superposition of two heat sources, a core (Figure 7) and a ring (Figure 8), results in a hybrid heat source: core + ring beam. The volumetric power distribution of this combined laser beam heat source, with a core power of 2000 kW and ring power of 1000 kW, is presented in Figure 9.

## 4. Thermal Phenomena

**Figure 10:**
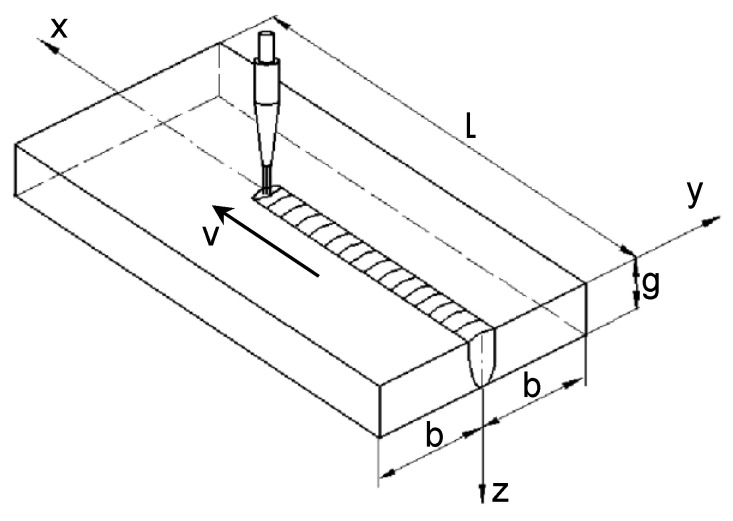
Schema of considered system. *L* is length of the specimen, *b* is width, *g* is thickness, whereas *v* is the heat source travel speed.

Figure 10 illustrates the three-dimensional formulation used to describe the thermal effects associated with bead-on-plate melting. The temperature field within the welded material is governed primarily by the magnitude and spatial distribution of the heat input, as well as by the heat source travel speed. When a highly focused heat source interacts with the surface, the material in this region undergoes intense melting and partial vaporization, which leads to the development of a keyhole. The numerical model takes into account phase transformations arising from changes in the material state [25,32], including the solid–liquid transformation and, at temperatures above the boiling point, transformations from liquid to vapor.

### 4.1. Governing Equations

The temperature field is obtained by the solution of energy conservation equation described in the following equation:

(2) $$\frac{\partial }{\partial x_{i}}\left(\lambda \frac{\partial T}{\partial x_{i}}\right)=C_{ef}\left(\frac{\partial T}{\partial t}+v_{i}\frac{\partial T}{\partial x_{i}}\right)-\tilde{Q}$$

where *T* = *T*(*x_i_,t*) is the temperature at point *x_i_*, *v_i_* is a velocity vector, *λ* = *λ*(*T*) is thermal conductivity, *C_ef_ = C_ef_*(*T*) is the effective heat capacity, which includes latent heat associated with material’s state change and latent heat of phase transformations in a solid state, and $\tilde{Q}$ is a volumetric heat source with laser beam and electric arc power distributions taken into consideration.

The initial condition $t=0:T=T_{0}$ and boundary conditions complete Equation (2), taking into account heat loss due to convection, radiation and evaporation:

(3) $$\Gamma :-\lambda \frac{\partial T}{\partial n}=\alpha (T|\Gamma -T_{0})+\varepsilon \sigma (T^{4}|\Gamma -T_{0}^{4})-q_{o}+q_{v}$$

where *α* is the convective coefficient, *ε* is the radiation coefficient, and *σ* is the Stefan–Boltzmann constant. Element $q_{o}$ is the heat flux towards the top surface of the welded element (*z =* 0) in the source activity field, while $q_{v}$ represents heat loss due to material evaporation in an area, where *T* ≥ *T_L_*, Γ is a boundary of the analyzed domain.

In the transient heat conduction model, changes of material state and the associated phase transformations are taken into account. For temperatures below the solidus, the effective heat capacity $C_{ef}$ is defined as the product of the material density and its specific heat in the solid phase. Within the temperature interval between the solidus and liquidus, the effect of melting is incorporated by adding the latent heat of fusion to the effective heat capacity [35]. In this region, a linear variation in the porosity factor is typically assumed:

(4) $$C_{ef}\left(T\right)=\rho_{SL}c_{SL}+\rho_{S}\frac{H_{L}}{T_{L}-T_{S}}\;\mathrm{for}\;T\in \left[T_{S};T_{L}\right]$$

where *T_s_* and *T_L_* are solidus and liquidus temperatures respectively, *H_L_* is the latent heat of fusion, and $c_{SL}\rho_{SL}=c_{S}\rho_{S}\left(1-f_{l}\right)+c_{L}\rho_{L}(f_{l})$ is the product of density and specific heat in the mushy zone.

For temperatures between the liquidus point and the boiling temperature of steel, the effective heat capacity is expressed as the product of the liquid-phase density and its corresponding specific heat. When the temperature rises above the boiling point, a linear interpolation of the liquid fraction is typically applied up to the maximum temperature $f_{l-g}\in \left[0;1\right]$. Under the assumption that the metal vapor pressure inside the keyhole is fully balanced by the shielding gas pressure, the effective heat capacity in this high-temperature range can be written in the following form:

(5) $$C_{ef}\left(T\right)=\rho_{L}c_{L}+\rho_{L}\frac{H_{b}}{T_{max}-T_{b}}\;\mathrm{for}\;T\geq T_{b}$$

where *T_b_* is a boiling point of steel, *T_max_* is the maximum temperature, and *H_b_* is the latent heat of evaporation.

Due to the highly turbulent motion of the molten metal within the fusion zone, a significantly higher effective thermal conductivity was assumed in this region compared to the solid part of the welded component. For temperatures $T<T^{*}=1273\;K$, the thermal conductivity λ=λ(T) was adopted according to the empirically determined relationship presented in [36]:

(6) $$\lambda \left(T\right)=59.92-0.0221T-5.4\cdot 10^{-5}T^{2}+4.3\cdot 10^{-8}T^{3},T\in [0,1000\;{}^{\circ}C]$$

In the temperature range 1273–$T_{S}$ [K], a constant value $\lambda_{\mathrm{const}}=\lambda (1273\;K)$ was assumed, whereas in the two-phase region (solid–liquid) a linear approximation of λ(T) was applied, as follows:

(7) $$\lambda (T)=\lambda (T^{*})+(\lambda (T_{L})-\lambda (T^{*}))\frac{T-T_{S}}{T_{L}-T_{S}}$$

In temperatures *T* > *T_L_*, thermal conductivity is set to *λ*(*T_L_*) = *λ_const_* = 117 [W/(mK)].

### 4.2. Numerical Solution

The solution formula of the heat transfer equation is obtained using the method of weighted residuals, which leads to the weak form of Equation (2):

(8) $$\int_{\Omega }\phi \left[\nabla \cdot \left(\lambda \nabla T\right)+\tilde{Q}\right]d\Omega =\int_{\Omega }\phi C_{ef}\dot{T}d\Omega$$

where ϕ is a weight function and $\dot{T}$ is the material derivative of the temperature.

The Petrov–Galerkin formulation [37,38] is used in Expression (8). Temperature in every node of FE mesh is the time function $T_{j}=T_{j}\left(t\right)$; thus, time integration of Equation (8) leads to the following formula:

(9) $$\begin{array}{l} \sum_{e}\left(K_{ij}^{e}+V_{ij}^{e}\right)\int_{t}\vartheta \left(t\right)T_{j}\left(t\right)dt+\sum_{e}M_{ij}^{e}\int_{t}\vartheta \left(t\right)\frac{\partial T_{j}\left(t\right)}{\partial t}dt=\\ =\sum_{e}S_{ij}^{e}\int_{t}\vartheta \left(t\right)Q_{j}^{e}\left(t\right)dt-\sum_{e^{\Gamma }}S_{ij}^{\Gamma }\int_{t}\vartheta \left(t\right){\tilde{q}}_{j}^{e}\left(t\right)dt \end{array}$$

where $K_{ij}^{e}$ is local conductivity matrix, $V_{ij}^{e}$ is the convection matrix, ϑ is the time weight function, $M_{ij}^{e}$ is the heat capacity matrix, $S_{ij}^{e}$ is the matrix of coefficients, $Q_{j}^{e}\left(t\right)$ is the vector of internal heat sources, $S_{ij}^{\Gamma }$ is the matrix of boundary coefficients, and ${\tilde{q}}_{j}^{e}\left(t\right)$ is the vector of boundary fluxes.

The final solution to the problem, after time integration, is expressed in the form

(10) $$\left(\beta K_{ij}+M_{ij}\right)T_{j}^{s+1}=\left[M_{ij}-\left(1-\beta \right)K_{ij}\right]T_{j}^{s}+\beta Q_{i}^{s+1}+\left(1-\beta \right)Q_{i}^{s}-\beta q_{i}^{*s+1}-\left(1-\beta \right)q_{i}^{*s}$$

where β is the time integration coefficient and s is the time increment.

Equation (10) is solved using the BiCGStab method [39].

The solution of the heat transfer equation with a convective term using the finite element method is difficult due to the presence of parabolic and hyperbolic terms in the equation. Stability of the solution is achieved by appropriate discretization of the system, reducing the characteristic dimension of the finite element. Meeting the discretization conditions with large Péclet numbers is extremely difficult, leading to oscillations in the calculation results. Elimination of solution oscillations is achieved by applying the Petrov–Galerkin formulation to the weighted residue criterion, which utilizes shifted weighting functions [38]. This method proceeds analogously to the Galerkin formulation, where weight functions equal element shape functions $w_{i}\left(x_{\alpha }\right)=\phi_{i}\left(x_{\alpha }\right)$, with the weighting functions modified by appropriate modifying terms ${\hat{w}}_{i}\left(\xi_{\alpha }\right)$, while maintaining all conditions regarding the constraints on the function carrier and the finite element integral:

(11) $$w_{i}\left(\xi_{\alpha }\right)=\phi_{i}\left(\xi_{\alpha }\right)+{\hat{w}}_{i}\left(\xi_{\alpha }\right)$$

The work uses polynomial weighting functions in which the modifying term is always of higher order than the shape function. For one-dimensional linear elements, the modifying term has the form

(12) $${\hat{w}}_{i}\left(\xi \right)=\xi_{i}\alpha_{i}P\left(\xi \right)$$

where $\xi_{i}=-1$ for node *i* and $\xi_{i}=1$ for node *i* + 1.

(13) $$\alpha_{i}=\alpha_{i}\left(Pe_{i}\right)=\cot h\left(Pe_{i}\right)-\frac{1}{Pe_{i}}$$

whereby

(14) $$Pe_{i}=\frac{{\left(Cef\right)}^{e}}{\lambda^{e}}h^{e}v_{i}$$

is the Péclet number for the finite element, while P(ξ) is the modifying function described by the relationship

(15) $$P\left(\xi \right)=k\left(1-\xi^{2}\right)$$

meeting the condition

(16) $$\left|\int_{-1}^{1}P\left(\xi \right)d\xi \right|=1$$

from which it follows that the coefficient k=3/4.

For normalized cubic elements (tri-linear), the weighting functions must be modified in the appropriate directions. Finally, the weighting function is described by the relationship

(17) $$w_{i}\left(\xi ,\eta ,\chi \right)=\left(\phi_{i}\left(\xi \right)+{\hat{w}}_{i}\left(\xi \right)\right)\left(\phi_{i}\left(\eta \right)+{\hat{w}}_{i}\left(\eta \right)\right)\left(\phi_{i}\left(\chi \right)+{\hat{w}}_{i}\left(\chi \right)\right)$$

## 5. Results and Discussion

Computer simulations are performed for melting flat sheets with a core source and separate ring source and a combined source (core + ring), taking into account the technological parameters used in the experimental studies (Table 1).

**Figure 11:**
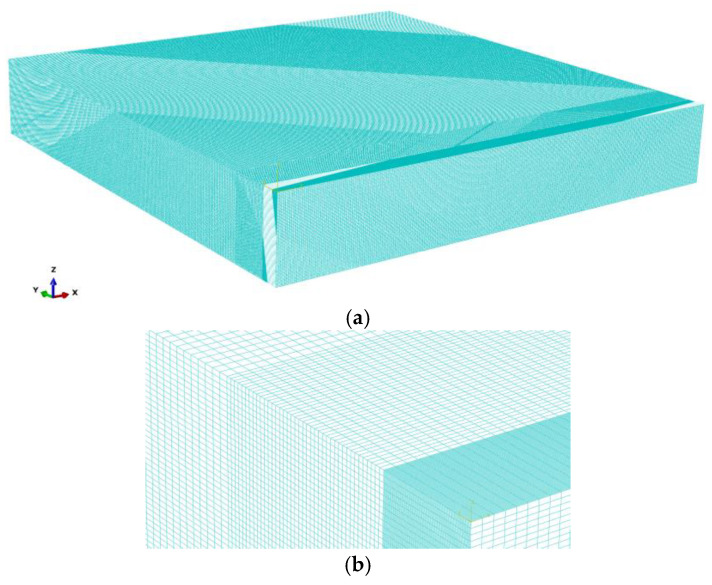
Finite element mesh used in calculations: (**a**) quarter of the entire mesh with visible plane of symmetry, (**b**) zoom in on the area encompassing the melted zone and the heat-affected zone.

Three simulations are performed with laser beam focusing: z = 0, the core beam diameter of 30 µm and ring beam diameter of 200 µm. Flat sheets used in the simulations are made of S355 steel with dimensions 250 × 50 × 4 mm. A grid with cuboid elements is assumed. The spatial step is set from 0.01 to 0.1 mm (Figure 11).

In the *y* direction, the spatial step is set to Δ*y* = 0.01 mm up to 1 mm (expected width of the fusion zone). Then, up to 3 mm (the expected width of the heat-affected zone), the mesh step is Δ*y* = 0.05 mm. In the base material, for smaller thermal gradients, the mesh step is set to Δ*y* = 0.1 mm. In the *x* direction, the mesh spatial step is set to Δ*x* = 0.1 mm. In the *z* direction, a constant spatial step is assumed: Δ*z* = 0.05 mm. Such a division of the finite element mesh generated 82 mln cuboid elements. Thermo-physical properties used in the numerical analysis are shown in Table 2.

**Figure 12:**
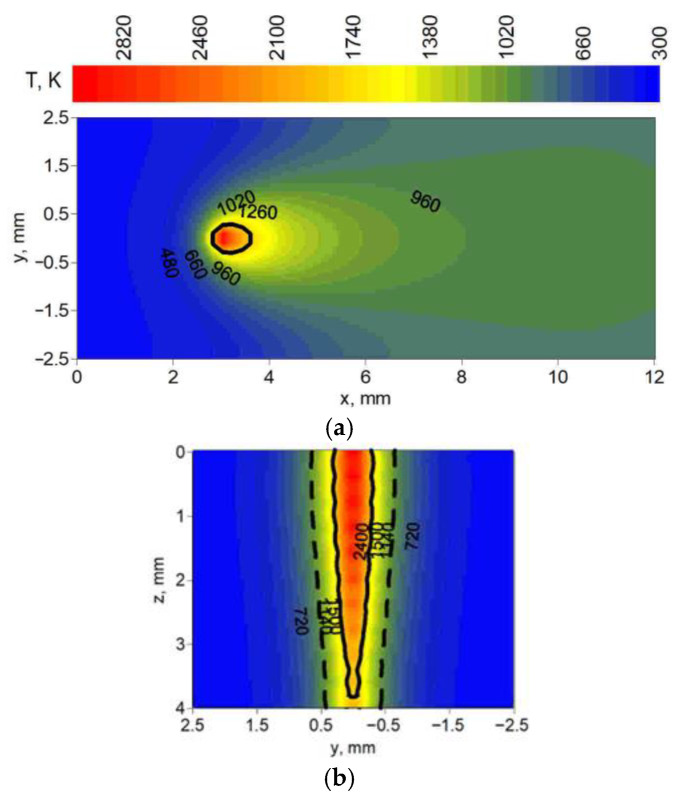
Temperature field: (**a**) at the top surface (z = 0), (**b**) in the cross-section (x = 3.1 mm) of the melted flat plate.

**Figure 13:**
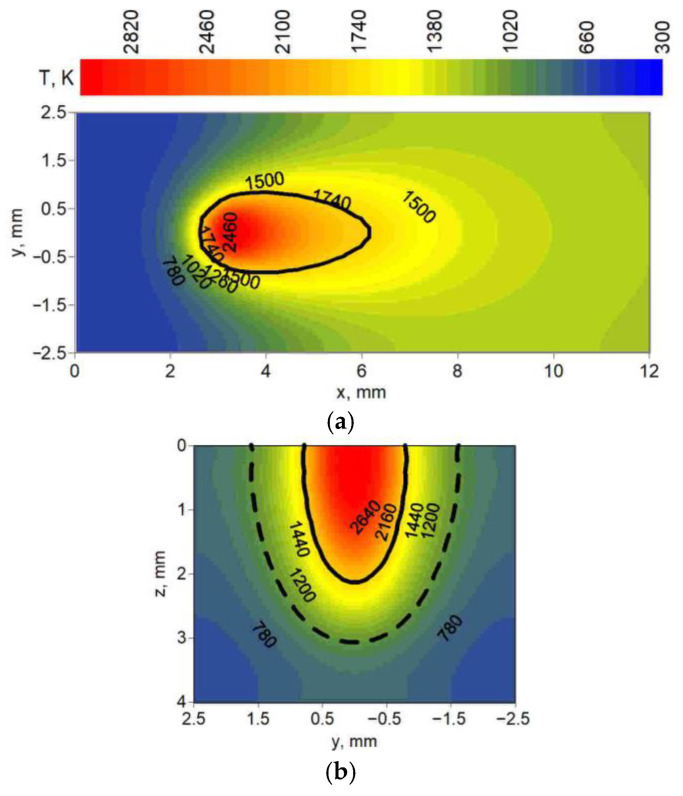
Temperature field: (**a**) at the top surface (z = 0), (**b**) in the cross-section (x = 3.4 mm) of the melted flat plate.

**Figure 14:**
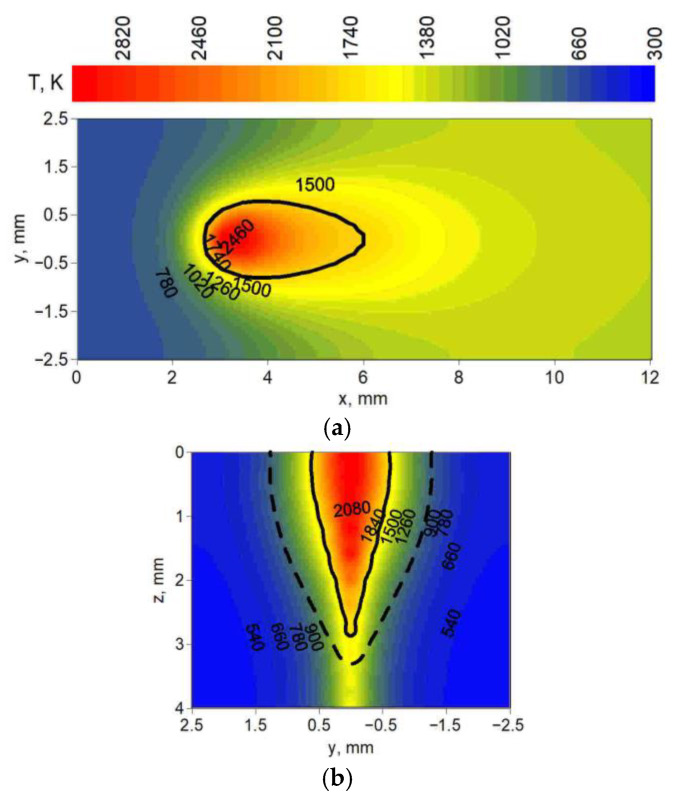
Temperature field: (**a**) at the top surface (z = 0), (**b**) in the cross-section (x = 3.6 mm) of the melted flat plate.

Figure 12, Figure 13 and Figure 14 present the temperature field at the top surface and in the cross-section of melted steel plates. In these figures, distributions of temperature show the differences in fusion zone geometry (solid line) and heat-affected zone geometry (dashed line) obtained in simulations. Cross-sections were analyzed for three yz planes located at different distances from the origin of the system in the direction of the melted seam (x axis). These distances were chosen for maximum fusion zone width and heat-affected zone width, taking into account the location of the center of the spot (z = 0, y = 0, x = 3 mm).

Figure 12 shows the temperature field for technological parameters used in test 6 (Table 1); Figure 13 shows the technological parameters used in test 14; and Figure 14 shows the technological parameters used in test 19.

Observing the predicted fusion zone and heat-affected zone, we can see that the core source has a very narrow, needle-like penetration (Figure 12). A wider ring source with lower power concentration, operating independently in the weld pool, results in less material penetration and wider characteristic zones (Figure 13). The combination of both core and ring sources allows for wider penetration (lower requirements for the set-up of welded elements) with deeper material penetration at a higher heat source speed compared to these sources operating separately (Figure 14).

**Figure 15:**
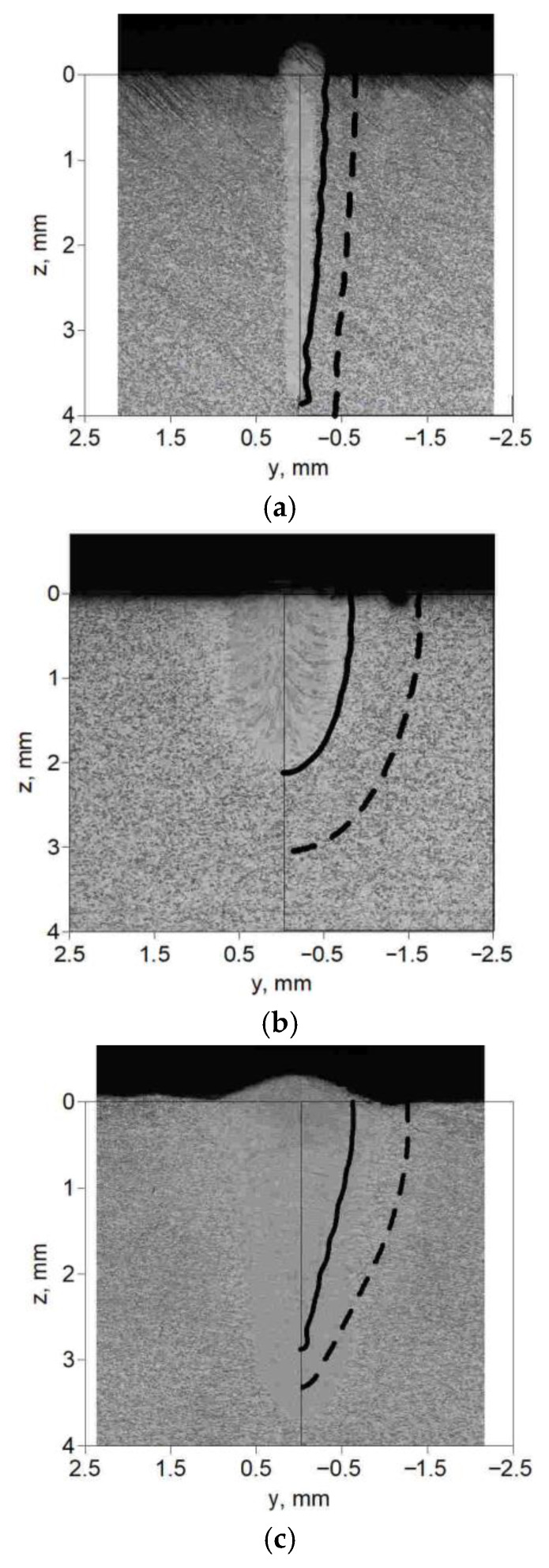
Comparison of predicted fusion zone (solid line) and heat-affected zone (dashed line) with the macroscopic image of the cross-section of a melted sheet using parameters in Table 1: (**a**) test no. 6, (**b**) test no. 14, (**c**) test no. 19.

In order to verify the correctness of the heat source models (core and ring), selected simulation results, i.e., the numerically estimated melted zone (continuous line) and the heat-affected zone (dashed line), are compared to the macroscopic cross-section image of experimentally performed melting tests of flat sheets made of S355 steel. Figure 15 shows the boundaries of characteristic zones marked on the photos of melted flat bars for test parameters 6 (a), 14 (b) and 19 (c).

From the analysis of Figure 15, it can be concluded that the predicted widths and shapes of the melted zone and heat-affected zone accurately reflect the actual experimental results. However, the predicted heat-affected zone for each case is slightly wider than the experimentally obtained zone. In the comparison presented in Figure 15c, we also see differences in the width of the predicted melted zone, given similar material penetration depths.

Quantitative comparison between numerical predictions and experimental measurements is performed for fusion zone width, penetration depth and heat-affected zone width. The mean relative error (MRE) and root mean square error (RMSE) are calculated for all analyzed cases. Table 3, Table 4 and Table 5 illustrate calculations of MRE and RMSE for the width of the fusion zone (FZ) and heat-affected zone (HAZ) and the material penetration depth (MPD).

(18) $$MRE=\frac{1}{n}\sum \left|\frac{x_{\alpha \;num}-x_{\alpha \;exp}}{x_{\alpha \;exp}}\right|\times 100\%$$

(19) $$RMSE=\sqrt{\frac{1}{n}{\sum \left(x_{\alpha \;num}-x_{\alpha \;exp}\right)}^{2}}$$

where *x_α_* is a measured (exp) or numerically predicted (num) dimension (*y* for width, *z* for depth).

The largest discrepancies are observed for the HAZ width, which is systematically overestimated by the numerical model. This behavior can be attributed to several simplifying assumptions adopted in the thermal model. In particular, the absence of melt pool convection and Marangoni-driven flow leads to reduced heat transport inside the liquid metal and increased conductive heat diffusion into the base material. Additionally, the model assumes constant absorptivity and simplified keyhole geometry, which may affect the lateral heat distribution. The use of effective thermal conductivity instead of fully coupled fluid flow modeling also contributes to smoothing of temperature gradients and widening of the predicted HAZ.

## 6. Conclusions

This study presents a mathematical and numerical framework for modeling the power density distribution of an innovative laser system composed of a single-mode core beam surrounded by a multimode ring beam. The use of the cylindrical-power-involution (CIN) model allowed accurate representation of both the concentrated single-mode heat input and the spatially distributed ring component. By superimposing these two analytical heat source formulations, a hybrid core + ring model was developed and implemented in three-dimensional transient thermal simulations of bead-on-plate laser melting of S355 steel plates.

Experimental measurements of the beam intensity profiles confirmed the distinct characteristics of the core and ring beams, while welding trials demonstrated their different influences on the geometry of the fusion and heat-affected zones. Numerical simulations reproduced these effects with good fidelity. The predicted fusion zone (FZ) and heat-affected zone (HAZ) boundaries closely matched the macroscopic cross-sections of experimentally welded samples for the selected test cases. The model correctly reflected the deep, narrow penetration associated with the core beam, the shallow and wide melt pool characteristic of the ring beam, and the synergistic effect of the combined source, which produced wider penetration at increased heat source travel speed. Minor discrepancies were observed mainly in the width of the predicted HAZ, which was slightly larger than the experimentally observed one.

Overall, the modeling approach proposed in this work provides a reliable tool for analyzing and predicting thermal phenomena in laser treatment processes that employ coaxial single-mode/multimode laser beams. The resulting hybrid heat source model captures the essential features of SM–MMR irradiation and can support optimization of process parameters, selection of process configurations, and further development of advanced multi-beam laser technologies.

Based on the performed simulations and experimental validation, additional detailed conclusions can be drawn:

1. *Accuracy of numerical prediction:* Quantitative comparison with experimental results shows good agreement for penetration depth and acceptable accuracy for fusion zone width. The mean relative error calculated for FZ width, HAZ width and penetration depth confirms that the proposed model can reliably predict characteristic zones for bead-on-plate SM–MMR laser melting of S355 steel, and simplifications of the model indicate that the total energy input is correctly represented.
2. *Observed discrepancies*: The numerical model slightly overestimates the heat-affected zone width and, in selected cases, predicts wider melt pools than observed experimentally. These differences are mainly related to model simplifications, including neglect of melt pool convection and Marangoni flow, simplified keyhole representation, constant absorptivity assumption, use of effective thermal conductivity instead of fluid flow modeling, and analytical representation of beam intensity distribution.
3. *Applicable range of the model*: The proposed model is particularly suitable for bead-on-plate laser melting, SM–MMR coaxial beam configurations, conduction–keyhole transition regimes, structural steels such as S355, process parameter studies and comparative analysis of core/ring interaction. The model is less suitable for full keyhole dynamic modeling, spatter prediction, fluid flow-dominated melt pools, highly unstable keyhole regimes, and pulsed laser processing.
4. *Process interpretation and control implications*: The simulations confirm that increasing ring beam power widens the fusion zone while maintaining penetration depth. This allows improved tolerance to joint fit-up and positioning accuracy. The SM–MMR configuration therefore provides an effective tool for controlling weld geometry by adjusting core-to-ring power ratio, ring diameter, travel speed, and total heat input. These parameters can be used to optimize weld width without excessive penetration or overheating.

Future research will focus on extending the model to include fluid flow effects in the weld pool, keyhole dynamics, and coupling between thermal and mechanical phenomena. Incorporating these phenomena is expected to enhance predictive capabilities, particularly for high-power and high-speed melting regimes characteristic of industrial laser processing.

## Figures and Tables

**Table 1:** Technological parameters used in the experiment.

Test Number	Travel Speed	Core Laser (Single-Mode)	Ring Laser (Multimode)
4	v = 5 m/min	Q = 1000 W	
5	v = 10 m/min	Q = 1000 W	
6	v = 15 m/min	Q = 1000 W	
13	v = 5 m/min		Q = 1000 W
14	v = 10 m/min		Q = 1000 W
15	v = 15 m/min		Q = 1000 W
19	v = 20 m/min	Q = 1000 W	Q = 1000 W
20	v = 20 m/min	Q = 1000 W	Q = 2000 W
21	v = 20 m/min	Q = 1000 W	Q = 3000 W
22	v = 20 m/min	Q = 1000 W	Q = 4000 W

**Table 2:** Thermo-physical parameters assumed in computer simulations [35,36].

Nomenclature	Symbol	Value
Solidus temperature	*T_S_*	1750
Liquidus temperature	*T_L_*	1800
Boiling point	*T_b_*	3010
Ambient temperature	*T_0_*	293
Specific heat of solid phase	*c_S_*	650
Specific heat of liquid phase	*c_L_*	840
Density of solid phase	*ρ_S_*	7800
Density of liquid phase	*ρ_L_*	6800
Latent heat of fusion	*H_L_*	270 × 10^3^
Latent heat of evaporation	*H_b_*	76 × 10^5^
Thermal conductivity of solid phase	*λ_S_*	45
Thermal conductivity of liquid phase	*λ_L_*	35
Convective heat transfer coefficient	*α*	50
Boltzmann’s constant	*σ*	5.67 × 10^−8^
Surface radiation emissivity	*ε*	0.5

**Table 3:** Error estimation for fusion zone.

Case	FZ Width Exp.	FZ Width Num	MRE	RMSE
Core beam			17.8%	0.03
z = 0	0.29 mm	0.30 mm		
z = 2.0 mm	0.15 mm	0.20 mm		
z = 3.8 mm	0.06 mm	0.07 mm		
Ring beam			19.8%	0.09
z = 0	0.70 mm	0.80 mm		
z = 1.0 mm	0.59 mm	0.71 mm		
z = 2.02 mm	0.20 mm	0.25 mm		
Core + Ring			15.9%	0.2
z = 0	0.95 mm	0.60 mm		
z = 1.0 mm	0.50 mm	0.50 mm		
z = 2.8 mm	0.09 mm	0.01 mm		

**Table 4:** Error estimation for heat-affected zone.

Case	HAZ Width Exp.	HAZ Width Num	MRE	RMSE
Core beam			29.9%	0.21
z = 0	0.90 mm	0.65 mm		
z = 2.0 mm	0.70 mm	0.44 mm		
z = 4.0 mm	0.40 mm	0.30 mm		
Ring beam			67.7%	0.51
z = 0	1.30 mm	1.6 mm		
z = 1.0 mm	1.10 mm	1.52 mm		
z = 2.02 mm	0.50 mm	1.21 mm		
Core + Ring			18.3%	0.18
z = 0	1.30 mm	1.35 mm		
z = 1.0 mm	1.20 mm	1.30 mm		
z = 2.8 mm	0.70 mm	0.40 mm		

**Table 5:** Error estimation for material penetration depth.

Case	MPD Exp.	MPD Num	MRE	RMSE
Core beam	3.8 mm	3.85 mm	1.3%	0.05
Ring beam	2.02 mm	2.09 mm	3.4%	0.07
Core + Ring	2.90 mm	2.91 mm	0.3%	0.01

## Data Availability

The original contributions presented in this study are included in the article. Further inquiries can be directed to the corresponding author.
